# Ligand‐Based Pharmacophore Mapping and Virtual Screening for the Search of Biguanide‐Like Molecules With Antidiabetic Potentials Targeting Liver Kinase B1

**DOI:** 10.1155/bri/8369459

**Published:** 2026-02-19

**Authors:** Rumman Reza, Md. Nazmus Samdani, Niaz Morshed, Raihana Haque Jebin, Imtiaz Ahmed, Md. Selim Reza

**Affiliations:** ^1^ Department of Pharmacy, University of Dhaka, Dhaka, Bangladesh, du.ac.bd; ^2^ Department of Pharmaceutical Technology, University of Dhaka, Dhaka, Bangladesh, du.ac.bd

**Keywords:** antidiabetic, biguanides, molecular docking, molecular dynamics simulation, T2DM

## Abstract

Type 2 diabetes mellitus (T2DM) is a state where the body’s glucose metabolism is compromised. AMP‐activated protein kinase, or AMPK, has an important part to play in glucose metabolism, and the liver kinase B1 (LKB1) protein functions as a major upstream kinase for AMPK activation, thereby making it appealing therapeutic targets for treating and preventing T2DM. Drug resistance cases for biguanides like metformin is a serious concern and pose great threat to treatment success for diabetic patients. Thus, the hunt for biguanide‐like small molecules with enhanced insulin sensitizing potentials is necessary. In the present study, interaction between LKB1 and biguanides such as phenformin, metformin, and buformin has been thoroughly assessed using computational tools. Ligand‐based pharmacophore mapping of 29,000 phytochemicals collected from NPASS database was carried out. The screening was conducted to hunt novel antidiabetic compounds targeting LKB1 pathway to improve insulin sensitivity in T2DM. Molecular docking of 31 phytochemicals with good pharmacophore fit scores was then carried out to identify hit compounds. ADMET analysis was also utilized to screen down compounds. dragmacidin D, dioncopeltine A, saussureamine C, and agelastatin D have good binding affinities and acceptable ADMET parameters. Molecular dynamics simulation was carried out to confer the stability of ligand–protein complex under simulated human body conditions. After 100 nanoseconds molecular dynamics simulation, the LKB1 protein complexed with compounds (saussureamine C and agelastatin D) was found to be stable. The results of the current study can be useful in developing antidiabetic medications with enhanced insulin sensitization activates superior to those available in market.

## 1. Introduction

One of the most alarming health issues in the world today is type 2 diabetes mellitus (T2DM) [1]. Currently, 6.28% of the world’s population is suffering from T2DM, which is expected to rise to 7.08% by 2030. With nearly a million deaths annually from diabetes, it ranks as the ninth most common cause of mortality globally [2]. Stroke, congestive heart failure, coronary artery disease, and peripheral vascular disease are some common comorbidities of T2DM [3]. T2DM is a chronic, noncommunicable condition that develops when our body’s ability to maintain blood glucose homeostasis is compromised [4]. A balanced system of hepatic glucose release and cellular glucose uptake keeps this homeostasis in place [5]. Glucose metabolism in adipocytes and skeletal muscle cells is also an important factor for this homeostasis [6]. Several signaling pathways are involved in the metabolism of glucose as well and one of them is the LKB1/AMPK pathway [7].

AMP‐activated protein kinase, or AMPK, being a major cellular energy sensor, plays a crucial role in metabolism of glucose. This protein kinase is activated by a couple of factors, such as the AMP: ATP ratio in cells, the LKB1 protein, and exercise [8]. AMPK facilitates cellular glucose uptake, fatty acid catabolism, and autophagy. On the other hand, it inhibits the synthesis of cholesterol, proteins, glycogen, and fatty acids [9]. LKB1 performs as an upstream kinase for AMPK [10]. Commonly known as a tumor suppressor, LKB1’s hypoglycemic activity is yet to be fully explored. In this study, we attempt to understand antidiabetic properties of LKB1. Indirect activators of AMPK are LKB1, calcium/calmodulin‐dependent protein kinase (CaMKKβ), transforming growth factor‐β (TGFβ‐activated kinase 1 (TAK1)), and so on [11].

Activation of AMPK through phosphorylation is more effective since it increases AMPK activity by more than 1000‐fold. On the other hand, activation by AMP via an allosteric mechanism only increases the activity by 5‐fold [12]. Since LKB1 activates AMPK through phosphorylating, it acts as a major upstream kinase for this pathway. Initially discovered to be modified in the rare genetic disorder Peutz–Jeghers syndrome (PJS), LKB1 codes for a 50 kDa serine/threonine kinase [13]. It regulates several cellular activities, including cancer, metabolism, and cellular polarity [8–10].

LKB1 gene is also known as serine/threonine kinase 11 gene [14]. LKB1, mouse protein 25 (MO25), and the STE20‐related adapter (STRAD) combine to create a trimolecular complex [15]. This trimolecular complex’s creation is essential for the LKB1‐AMPK signaling pathway. Fyn kinases are reported to decrease the phosphorylation of AMPK by inhibiting the activation of LKB1 [16]. Researchers have conducted a study where they used a group of hypomorphic LKB1 mice, in which skeletal muscle LKB1 was removed and LKB1 protein in the whole body was reduced by 70%–80% and the results were compared with a group of whole‐body AMPK (α1 and α2) knockout mice and AMPKα2 inactive transgenic mice. According to the findings, glucose transport in skeletal muscle was partially impeded in the first group, whereas it was normal in the second. Therefore, it can be said that LKB1 is essential for the metabolism of glucose and that, in the absence of it, glucose metabolism may be disrupted, leading to diseases like diabetes.

Several drugs have been found to help activate the LKB1‐mediated AMPK pathway, therefore are being used as therapeutic agents for treating diabetes [5, 17]. Biguanides are the most popular antidiabetic agent for the treatment of T2DM. Various studies have shown that, both in vitro and in vivo, metformin stimulates AMPK. Additionally, it has been found that metformin’s activation of AMPK is necessary for hepatocytes to produce less glucose and burn more fat, as well as for skeletal muscle to absorb more glucose [18]. Thus, in this study, biguanides like metformin, phenformin, and buformin were used as standards to hunt for similar small molecules that will act as agonists of LKB1 and eventually help activate LKB1‐mediated AMPK pathway.

In the present study, the role of LKB1 in T2DM has been elucidated. Ligand‐based pharmacophore mapping was carried out to search biguanide‐like small molecule agonists of LKB1 protein. 29,000 natural phytochemicals from NPass database were used to build natural library of compounds. Pharmacophore mapping was carried out using metformin, buformin, and phenformin as control ligands. Molecular docking and ADMET analysis of the proposed hit compounds were utilized in this work to filter out drug‐like leads. Molecular dynamics (MD) simulation studies were done to evaluate the stability of ligand–protein complexes under human body conditions. The main aim of this research is to search novel antidiabetic drugs that can prove to be beneficial in the treatment of T2DM in cases where current drug is ineffective due to rising drug resistance instances.

## 2. Methodology

### 2.1. Evaluation of Disease–Gene Association Between LKB1 and Diabetes Mellitus Using DisGeNET

The current study examined the relationship between diabetes mellitus and LKB1 using the DisGeNET web server [19]. Diabetes mellitus was included as a search term in the web tool’s search section. The default search parameter led to the generation of the disease summary. A number of genes linked to diabetes mellitus were tabulated in the subsection on disease–gene associations along with disease specificity and pleiotropy indices.

### 2.2. Investigation of Protein–Protein Interaction Network (PPI) Using STRING

Using the STRING platform, a protein–protein network was constructed for LKB1 [20]. Target protein was entered into the web tool’s designated search field. For search results, the target organism was set to *Homo sapiens*. The web tool produced a protein network that appeared like colored nodes and displayed the first line of a list of proteins that interacted with the target proteins.

### 2.3. Validation of Molecular Docking Protocol

In this study, the PDBID of LKB1 protein, 2WTK, was used. The PDB file of the protein was downloaded and opened using BIOVIA Discovery Studio. The protein structure was cleaned by removing associated ligands. The cleaned protein structure was saved in PDB format. The original PDB file was used as reference structure. Structure data file of bound ligand phosphoaminophosphonic acid‐adenylate ester was retrieved from PubChem. Docking of ligand and cleaned protein was carried out in AutoDock Vina panel of PyRx (version 1.1). The root mean square deviation (RMSD) value of the reference structure and redocked protein structure was evaluated using PyMOL. To determine the RMSD between two or more molecular structures, PyMOL provides a number of functions. For this, align, super, and pair fit are the most often used commands. The align command aligns two structures in a series and then superimposes them to reduce the respective atoms’ RMSD. The external window displays the RMSD value.

### 2.4. Preparation of Target Protein

The crystal structure of human LKB1 protein (PDB code: 2wtk) (resolution: 2.90 Å) was downloaded from Protein Data Bank (https://www.rcsb.org). For performing molecular docking simulation, LKB1 was prepared by removing water molecules and adding polar hydrogen using PyMOL (version 2.6.2).

### 2.5. Docking Studies of Target Macromolecule With Biguanides

The chemical structures of metformin, phenformin, and buformin were downloaded from PubChem (https://pubchem.ncbi.nlm.nih.gov/) in SDF format. A molecular docking simulation was conducted for the biguanides with prepared LKB1 protein macromolecule using the AutoDock Vina program in PyRx software (Scripps Research Institute, La Jolla, CA, USA). For pharmacophore modeling, the receptor–ligand complex was saved as a PDB file after molecular docking simulation.

### 2.6. Pharmacophore Generation of Biguanides and LKB1 Docked Complex and Pharmacophore Mapping to Screen NPass Natural Compound Database of 29,000 Phytochemicals

Pharmacophore model of receptor–ligand complex for metformin and LKB1 was generated using LigandScout software (Inte:Ligand GmbH, Vienna, Austria). The pharmacophore model was then copied to screening window in the software. A database of 29,000 natural compounds downloaded from the NPass server (https://www.npass.com) was generated for virtual screening with LigandScout (version 4.4) [21].

For screening purposes, every aspect of the produced pharmacophore was employed. From the NPASS database, a library of 29,000 natural chemicals was obtained [22]. The SDF format of the structures found in the compound library was downloaded. Next, the entire library was converted to an IDB file format. Subsequently, the pharmacophore that was produced was aligned with the 29,000 natural molecules found in the NPASS chemical library. Afterward, molecules possessing same pharmacophore characteristics were identified using the default settings [21].

### 2.7. Molecular Docking of Leads With LKB1 Protein

We used BIOVIA Discovery Studio 2019 (DS) to remove additional protein chains and water atoms from LKB1. A PDB structure file was generated for the cleaned structure of the protein. Using PyRx, the active compounds were blindly docked against LKB1 to determine their binding affinities. PyRx software (version 1.1) was used to access the receptor protein [23]. The structure data files (SDF) for 52 different chemicals were acquired from PubChem. The SDFs of the selected compounds were allocated the ligand label and used as inputs. LKB1 and 31 compounds were docked using the AutoDock Vina platform of the PyRx programme [24]. The ligand–protein interaction was visualized using BIOVIA Discovery Studio 2019 [25]. The affinities of the lead compounds for binding were contrasted with those of the control ligand metformin.

### 2.8. ADMET Prediction of Phytochemicals

ADMET analysis of the top 20 compounds with high binding affinity towards LKB1 was carried out using SwissADME web server. To assess if natural compounds were drug‐like, the following criteria were used: ESOL class, CYP inhibitor, BBB permeant, Lipinski rule, GI absorption, and bioavailability grade using SwissADME system [26]. The ligand structure data files were gathered from PubChem. The files that were downloaded were uploaded one after the other to the web server of SwissADME. The generating process produced tabulated results.

### 2.9. Estimation of Oral Bioavailability

The bioavailability radar profiles of them were collected from the SwissADME website in order to determine the drug‐likeness of the top 5 compounds. The canonical smile IDs for these five compounds were first collected from the PubChem database. Subsequently, the canonical smile ID was uploaded to the SwissADME website for each substance. The website showed previously recorded information on the compound that was requested. The bioavailability part provided the radar illustrates.

### 2.10. Interaction Studies of Docked Complexes

The docked complexes of ligands with good binding affinities and good ADMET were studied further by analyzing the interaction of ligand with amino acid residues of target protein. The docked complexes of CID 24862587, CID 9998735, CID 177418, CID 185971 CID 15000037, and CID 24862637 were saved as Protein Data Bank files using PyMOL software. The PDB files were opened separately in Discovery Studio software. Ligand–protein interaction 2D diagram was generated by using the ligand interaction option in the software. Furthermore, the pharmacophore of ligand–protein docked complexes was generated in LigandScout software to study the pharmacophore models.

### 2.11. MD Simulation Studies

**FIGURE 1:**
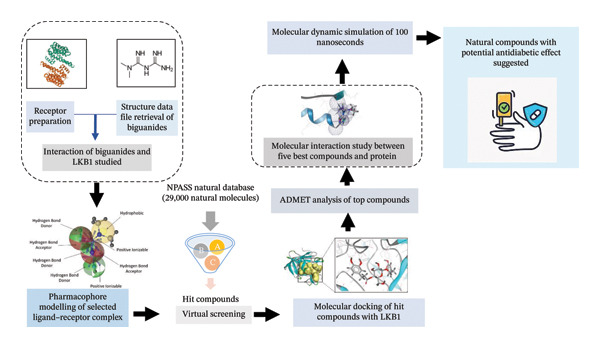
Detailed stepwise graphical representation of the methodology adopted in the present study.

We used the “Desmond v3.6 Programme” in Schrödinger (academic version) on a Linux operating system to run 100 ns MD simulations to assess the binding stability of phenformin (control) and some of the selected potential hit compounds. The dynamics system was an orthorhombic periodic boundary box with a particular volume and a distance of 10 Å assigned in the predefined TIP3P water model. Na+ and Cl−, the appropriate ions, were added to this solvated solution at a concentration of 0.15 M to achieve equilibrium. Using the OPLS3 force field settings and the Desmond module’s default protocol, the system was minimized and relaxed [27]. With recording intervals of 100 ps, an NPT ensemble maintained 300 K and one atmospheric (1.01325 bar) pressure. The Desmond module’s simulation interaction diagram (SID), available in the Schrödinger package, has been utilized to analyze the findings. Using the RMSD and RMSF, the stability of the complex structure has been evaluated through the analysis of the simulation trajectory. The entire workflow followed in the current study is depicted in Figure 1.

## 3. Results

### 3.1. Disease–Gene Association Between LKB1 and Diabetes Mellitus

The DisGeNET platform has one of the largest freely available datasets of genes and variants associated with human illnesses. The online server integrates data from expert‐curated sources, GWAS libraries, animal models, and academic literature. Controlled vocabularies and community‐driven ontologies are used to uniformly label DisGeNET data. Moreover, several distinct markers are provided to assist in ranking the associations between genotype and phenotype. The gene–disease association score for LKB1 gene with selective diseases are as follows: diabetes mellitus (0.040), noninsulin‐dependent diabetes mellitus (0.040), hyperglycemia (0.030), diabetic nephropathy (0.020), and insulin resistance (0.20).

AMPK is a serine/threonine protein kinase. Activation of AMPK pathway leads to glucose utilization. It is comprised of three different subunits α, β, and γ, forming a well‐conserved heterotrimeric complex [28]. The α subunit (α1 and α2) is catalytic and it combines with the regulatory subunits β (β1, β2) and γ (γ1, γ2, γ3), all of which are encoded by separate genes. This complex ultimately yields 12 heterotrimeric combinations. Although the α1 subunit of AMPK is placed in non‐nuclear fractions, the α2 and β subunits are found in both the nucleus and the cytoplasm. Moreover, the β1 subunit can bind to the plasma membrane through a myristoylation site on the N‐terminal. It has been also found that AMPK with α1 subunits is less sensitive to AMP.

**FIGURE 2:**
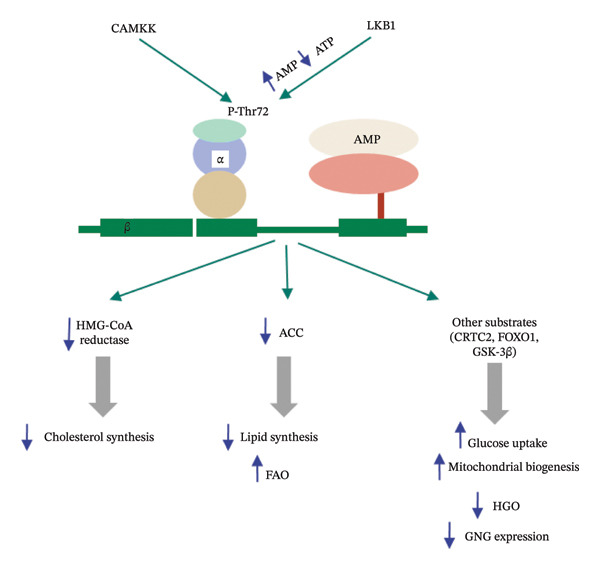
Activation of AMPK pathway by different upstream kinases and subsequent beneficial effects. LKB1 helps to activate AMPK pathway which has several potential benefits such as increase in glucose uptake and reduction in cholesterol synthesis. This ultimately accentuates insulin sensitizing activities.

The activation of AMPK is regulated by several direct/indirect factors. The direct activators of AMPK include AMP, ADP, A‐769662, AICAR (5‐aminoimidazole‐4‐carboxamide ribose), and salicylate. Among these, AMP and ADP are the physiological direct activators while AICAR, A‐769662, and salicylate are the pharmacological direct activators. The direct activators either allow the catalytic α subunit to be phosphorylated or activated at Thr172 by binding to it or for allosteric activation bind to the regulatory γ‐subunit. LKB1 accentuates the activity of alpha subunit of AMPK. The interaction is important for the activation of AMPK‐mediated pathway which in turn facilitates glucose uptake. The pathway of interaction between LKB1 and AMPK is annotated in Figure 2.

### 3.2. Protein–Protein Network Analysis Using STRING

**FIGURE 3:**
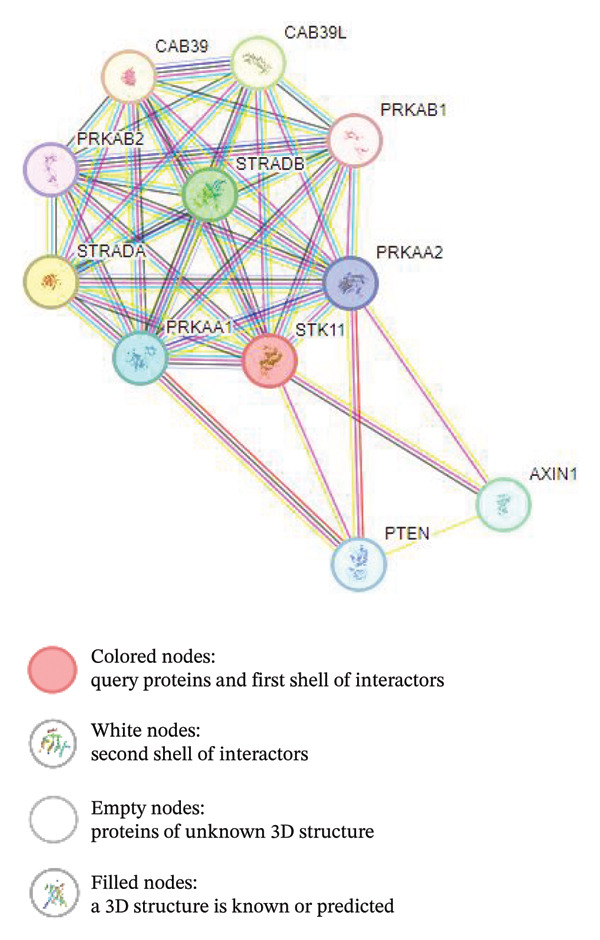
Protein–protein network of LKB1 (also known as STK11) protein generated using STRING platform. LKB1 forms first line of interaction with PRKAA2, PRKAB1, PRKAB2, PTEN, PRKAA1, AXIN1, CAB39, STRADB, and CAB39L.

In molecular biology, STRING is an online resource and biological library for known and predicted protein–protein interactions [20]. The STRING database contains data from a variety of sources, such as experimental data, computer prediction methods, and public text collections. Tumor suppressor serine/threonine protein kinase STK11 regulates the activity of members of the AMPK family, which in turn affects a number of functions including cell metabolism, polarity, apoptosis, and the response to DNA damage. STK11 acts by phosphorylating the AMPK family proteins’ T‐loop, which increases the activity of the proteins: PRKAA1 (AMPK catalytic domain 1), PRKAA2 (AMPK catalytic domain 2), BRSK2, MARK1, MARK3, MARK2, MARK4, SIK1, BRSK1, NUAK1, NUAK2, SIK2, MARK3, SIK3, and SNRK are all phosphorylated, but not MELK. The phosphorylated proteins are not members of the AMPK family, including STRADA, PTEN, and p53/TP. The protein–protein network diagram is presented in Figure 3.

### 3.3. Docking Protocol Validation

The docking protocol was validated prior to screening operations. Docking validation is the process of evaluating the dependability of a docking methodology by comparing its output to known experimental data. Usually, this is done by redocking with a co‐crystallized ligand to check RMSD and successful binding pose prediction. Comparing the RMSD between the native ligand’s crystallographic pose and the redocked pose is a common technique; a low RMSD (preferably < 2 Å) denotes successful validation. The RMSD of the native pose and redocked ligand pose was found to be 1.64 Å which is less than 2 Å. The docked pose of both the native and redocked protein structures is given in Supporting Figure 1.

### 3.4. Molecular Docking of Biguanides With LKB1 Protein

Using molecular docking, the potential interactions between the phytochemical components and LKB1 were examined in this study. A vital tool in computer‐assisted drug design and structural molecular biology is molecular docking. Predicting the main binding mode(s) of a ligand with a protein that has a known three‐dimensional structure is the aim of ligand–protein docking [29]. A shared nitrogen atom unites two guanidine groups to form a class of chemicals known as biguanides [30]. Due to two imino and three amino groups in tautomerism, they are all polar and hydrophilic molecules that are very soluble in aqueous solutions [31]. The pharmacokinetic and pharmacodynamic characteristics of each of them are caused by certain chemical differences between them [32]. In the present study, biguanides (metformin, phenformin, and buformin) were docked with LKB1 protein to study the effect of these biguanides on the target protein. Phenformin, buformin, and metformin showed binding affinities of −7.1 kcal/mol, −5.4 kcal/mol, and −4.7 kcal/mol, respectively.

### 3.5. Pharmacophore Model Generation With Biguanide–LKB1 Docked Complex and Ligand‐Based Pharmacophore Screening

**FIGURE 4:**
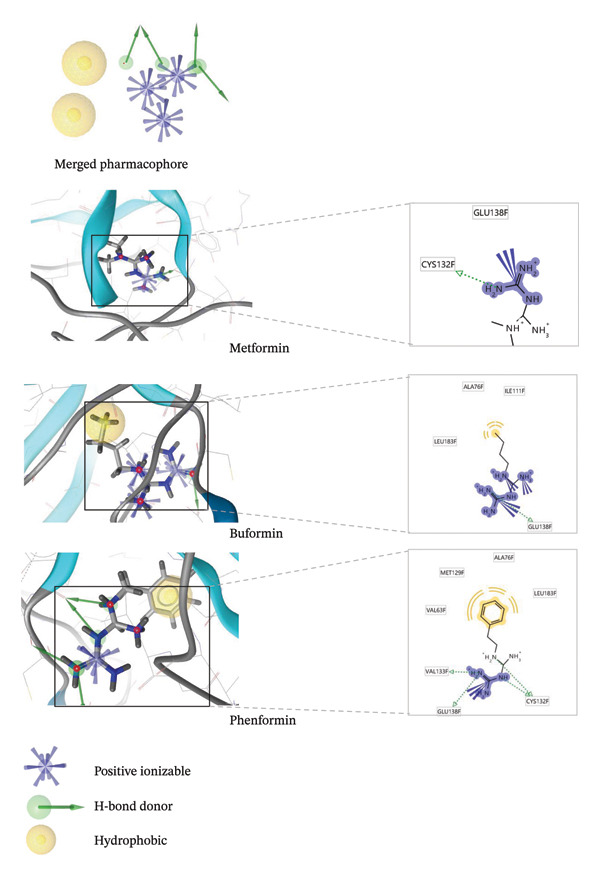
Pharmacophore of LKB1 protein with biguanides (metformin, buformin, and phenformin). Interaction generated using LigandScout software.

Pharmacophore modeling is typically used in computer‐aided drug design. The mapping technique is used in defining the appropriate pharmacophoric characteristics of a drug molecule that are required to have a certain pharmacological effect and to clearly define the links between structure and activity. To create new, more potent compounds that fit the model, a well‐developed pharmacophore model that preferably includes details about the size of the receptor binding cavity may be used [21, 33]. Pharmacophore generation of the docked structures of biguanides with LKB1 protein allowed for the investigation of the interaction between ligands and protein macromolecule. In Figure 4, it is seen that metformin formed hydrogen bond with CYS132 residue of LKB1. Buformin acted as a hydrogen bond donor towards GLU138 amino acid residue of LKB1. Phenformin formed four hydrogen bonds with VAL133, GLU138, and CYS 132 residues. Hydrophobic interactions were observed for buformin and phenformin.

The three biguanides were used to create the ligand‐based pharmacophore model. Subsequently, the ligands were grouped based on their pharmacophore RDF similarity and their chemical properties were combined to create the merged pharmacophore. Based on atom overlap and pharmacophore fit, the pharmacophore with the highest score was selected. Its characteristics include one H‐bond donor and two hydrophobic interactions. Next, using the default settings, the pharmacophore was run against the collection of 29,000 natural chemicals from NPass database [21]. Using the combined pharmacophore’s properties, the putative agonists of LKB1 were identified. The hit rate was 0.0018%, with 52 hit compounds in total.

### 3.6. Molecular Docking of Leads With LKB1

The hit compounds were docked with LKB1 protein. The protein was cleaned by removing water atoms and heteroatoms. Then, the hit compounds with good pharmacophore fit scores were docked with the cleaned protein structure of LKB1. The top 10 compounds had binding affinities ranging from −11 to −8.4 kcal/mol. The next 10 compounds had binding affinities ranging from −8.4 to −7.4 kcal/mol. All of these compounds had binding affinities greater than that of control ligands (metformin, buformin, and phenformin). The binding affinities of the hit compounds with LKB1 protein are enlisted in the Supporting Table 1.

### 3.7. ADMET Analysis of Phytochemicals

The early stages of drug development have relied heavily on the estimation of pharmacokinetic parameters to guide hit‐to‐lead and lead‐optimization initiatives [34]. Drug discovery applicants have actively sought molecular modeling techniques to find patterns in ADMET data and turn them into knowledge, given the exceptional complexity of the present R&D model. The field has developed in tandem with chemoinformatics, which has progressed from conventional chemometrics to sophisticated machine learning techniques. In the present study, ADMET properties were assessed for the top 20 hit compounds using SwissADME web server. The ADMET parameters for the top 20 hit compounds are enlisted in Table 1. All the parameters as collected from SwissADME web server are enlisted in Supporting Table 2. The molecules with CID 185971, CID 9998735, CID 177418, CID 24862637, CID 24862587, CID 14162516, and CID 10387020 had zero violations of Lipinski rule of five. The compounds CID 72495 and CID 10794221 showed one violation of Lipinski rule. Metformin, buformin, and phenformin have good GI absorption profiles. Also, these compounds showed zero violation of Lipinski’s rule of five.

**TABLE 1:** ADMET properties of compounds generated using SwissADME web server.

Molecule	MW	ESOL class	GI absorption	BBB permeant	PGP substrate	Lipinski violations
CID_15000037	532.39	Moderately soluble	Low	No	No	2
CID_10985632	791.98	Poorly soluble	Low	No	Yes	3
CID_44448166	649.29	Moderately soluble	Low	No	No	3
CID_71463188	812.57	Moderately soluble	Low	No	Yes	3
CID_11204813	708.02	Poorly soluble	Low	No	No	2
CID_185971	379.45	Moderately soluble	High	No	Yes	0
CID_9998735	362.42	Very soluble	High	No	No	0
CID_177418	327.13	Very soluble	High	No	Yes	0
CID_52951296	704.45	Moderately soluble	Low	No	No	2
CID_90681741	582.79	Moderately soluble	Low	No	No	2
CID_72495	627.96	Moderately soluble	Low	No	No	1
CID_24862637	394.42	Highly soluble	Low	No	No	0
CID_24862587	365.42	Very soluble	High	No	No	0
CID_11671852	641.1	Moderately soluble	Low	No	No	2
CID_10794221	641.95	Soluble	Low	No	No	1
CID_14162516	271.36	Soluble	High	No	Yes	0
CID_10387020	413.31	Moderately soluble	High	No	Yes	0
CID_71746447	445.51	Very soluble	Low	No	No	2
CID_3011701	783.14	Poorly soluble	Low	No	Yes	3
CID_44583735	657.99	Soluble	Low	No	No	2
Metformin (control)	129.16	Highly soluble	High	No	No	0
Buformin (control)	157.22	Very soluble	High	No	No	0
Phenformin (control)	205.26	Very soluble	High	No	No	0

### 3.8. Prediction of Oral Bioavailability

**FIGURE 5:**
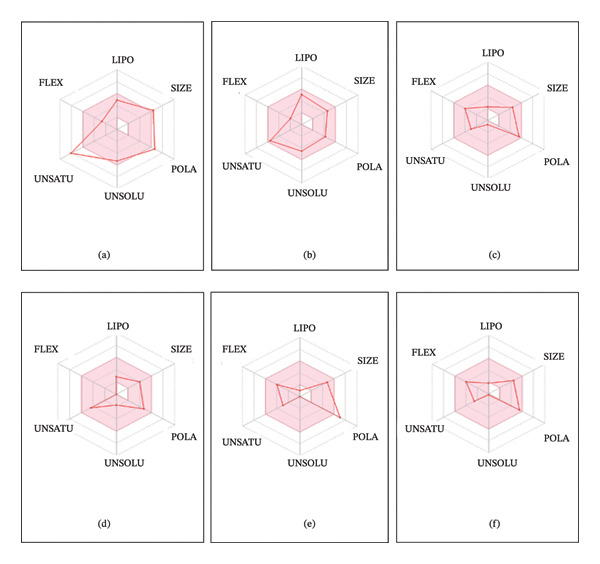
Bioavailability radar images of hit compounds ((a) CID 15000037, (b) CID 185971, (c) CID 9998735, (d) CID 177418, (e) CID 24862587, and (f) CID 24862637). The radar images have been retrieved from SwissADME web server.

Bioavailability radar provides a graphical description of the requirements for an oral biologically active medicament and allows for a fast evaluation of a molecule’s drug‐likeness. To assess the oral bioavailability potential of the hits, the bioavailability radar for each compound was gathered from SwissADME. The relevant ADME data for every compound was shown following the entry of the molecules’ smile IDs. The bioavailability component of the ADME information provided the radar images of bioavailability. Figure 5 shows the bioavailability radar profiles of the top 6 compounds. Also, the bioavailability radar images of control compounds (metformin, buformin, and phenformin) are depicted in Supporting Figure 2 for comparison. Metformin and buformin have well‐balanced bioavailability parameters, while for phenformin, unsaturation parameter is slightly over the edge compared to other parameters.

The compound parameters are within the maximum limit range of the physicochemical properties that control a drug’s bioavailability according to the bioavailability radar pictures [34]. The radar is shown as a hexagon, where each vertex represents a feature of the drug’s bioavailability. The ideal range for each property such as size (MW between 150 and 500 g/mol), lipophilicity (XLOGP3) between −0.7 and +5.0, saturation (fraction of carbon atoms in the sp3 hybridization not lower than 0.25), solubility (log S not higher than 6), polarity (TPSA between 20 and 130 Å), and flexibility is represented by the pink area in the radar data gathered from SwissADME [34]. agelastatin D (CID 177418), pulchellamine B (CID‐24862637), dioncopeltine A (CID 185971), and saussureamine C (CID 9998735) showed a good balance of all these parameters. Dragmacidin D (CID 15000037) showed slightly increased saturation but was within limit and portrayed well‐balanced bioavailability parameters. For pulchellamine A (CID 24862587), polarity (TPSA) was marginally over the edge but the other parameters were well within boundary.

### 3.9. Interaction Study of Ligands With LKB1

**FIGURE 6:**
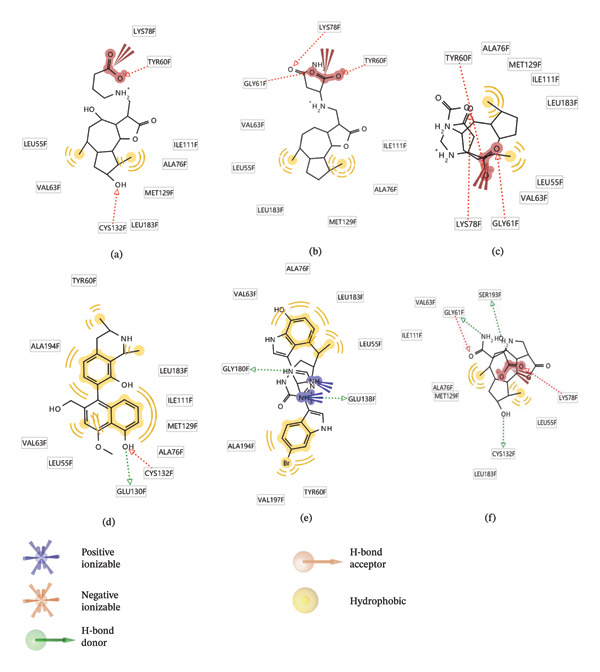
Protein–ligand pharmacophore between LKB1 with selected compounds and chemical features involved in interaction. (a) CID 24862587, (b) CID 9998735, (c) CID 177418, (d) CID 185971, (e) CID 15000037, and (f) CID 24862637.

The compounds showing good ADMET and oral bioavailability properties were analyzed for interaction with LKB1 protein (Table 2). The compounds formed hydrogen and hydrophobic bonds with the target protein. Dragmacidin D showed the lowest binding affinity score among the filtered compounds which is −11 kcal/mol. The strong bond formation was due to hydrogen bond formation between dragmacidin D and the protein in residues CYS132, GLY180 and GLU180, with further bonding of hydrophobic bonds with residues LEU183, LEU55, ALA76, VAL63, and MET129. Dioncopeltine A (−8.9 kcal/mol) and agelastatin D (−8.7 kcal/mol) showed similar hydrogen and hydrophobic bond formation with the protein. Sausseramine C (−8.7 kcal/mol) and pulchellamine B (−8.3 kcal/mol) formed hydrogen bond with SER59, TYR60, and GLY61 residues which also showed high binding affinity with the protein. It indicates that these residues are important to induce LKB1 protein. Pulchellamine A (−8.2 kcal/mol), trypargine (−8 kcal/mol), and gelliusine (−7.9 kcal/mol) also showed lower binding affinity score than the biguanide controls due to formation of hydrogen and hydrophobic bonds. Table 2 shows the amino acid residues of LKB1 with which the selected compounds form hydrogen bonds and hydrophobic interactions. Pharmacophore of hit compounds with LKB1 protein was generated using LigandScout software and is depicted in Figure 6. The 3D and 2D interaction diagrams of ligand–LKB1 docked complexes are shown in Figures 7 and 8, respectively.

**TABLE 2:** The interacting amino acid residues of LKB1 with top ligands showing hydrogen bonds and hydrophobic interactions.

Compound ID	Compound name	Binding affinity (kcal/mol)	Type of bond	Interacting residues
CID 15000037	Dragmacidin D	−11	Hydrogen	CYS132, GLY180, GLU138
			Hydrophobic	LEU183, TYR60, LEU55, ALA76, VAL63, MET129, LYS78, ALA194, VAL197

CID 185971	Dioncopeltine A	−8.9	Hydrogen	CYS132, GLY180
			Hydrophobic	LEU183, TYR60, LEU55, ALA76, VAL63, MET129, LYS78, ALA194

CID 9998735	Saussureamine C	−8.7	Hydrogen	SER59, TYR60, GLY61, LYS78
			Hydrophobic	LEU183, LEU55, ALA76, VAL63, MET129, CYS132, ILE11

CID 177418	Agelastatin D	−8.7	Hydrogen	LEU55, GLU138, CYS132
			Hydrophobic	LEU183, VAL63

CID 24862637	Pulchellamine B	−8.3	Hydrogen	SER59, TYR60, GLY61, GLU130, SER193, ALA194
			Hydrophobic	LEU55, LEU183, VAL63, MET129

CID 24862587	Pulchellamine A	−8.2	Hydrogen	SER59, TYR60, GLY61, LYS78, SER193
			Hydrophobic	LEU183, VAL63, ALA76, MET129, LEU55

CID 14162516	Trypargine	−8	Hydrogen	CYS132, VAL133, LEU55
			Hydrophobic	LEU183, VAL63, ALA76, MET129, LYS78

CID 10387020	Gelliusine E	−7.9	Hydrogen	SER59, TYR60, GLY180, ASN181
			Hydrophobic	LEU55, LEU183, VAL63, ALA76, ALA194

CID 8249	Phenformin	−7.1	Hydrogen	LEU55, CYS132, VAL133
			Hydrophobic	VAL63, ALA76, MET129, LYS78

CID 2468	Buformin	−5.4	Hydrogen	LEU55, CYS132, VAL133
			Hydrophobic	VAL63, ALA76, LEU183

CID 4091	Metformin	−4.7	Hydrogen	LEU55, CYS132, VAL133

FIGURE 7Three‐dimensional interaction images of hit compounds docked with LKB1 protein ((a) CID 15000037, (b) CID 185971, (c) CID 9998735, (d) CID 177418, (e) CID 24862587, and (f) CID 24862637). The images have been generated using BIOVIA Discovery Studio.

**Figure:**
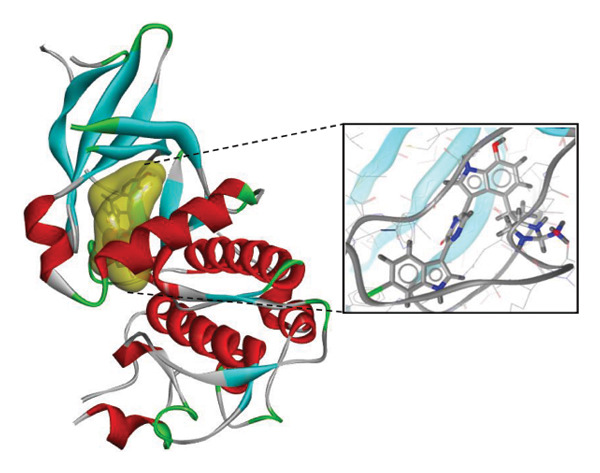
(a)

**Figure:**
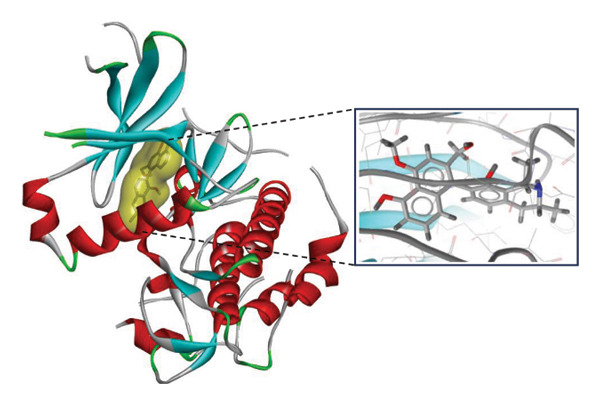
(b)

**Figure:**
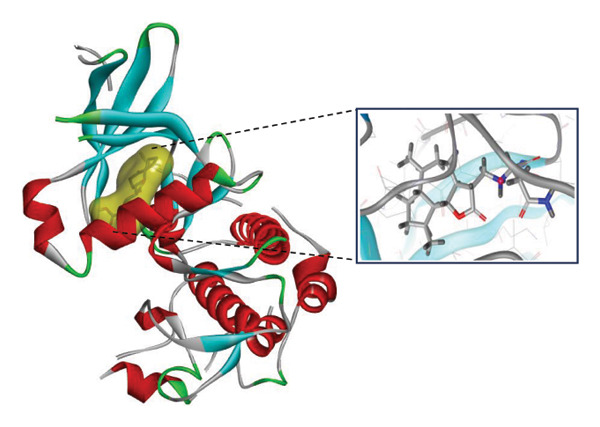
(c)

**Figure:**
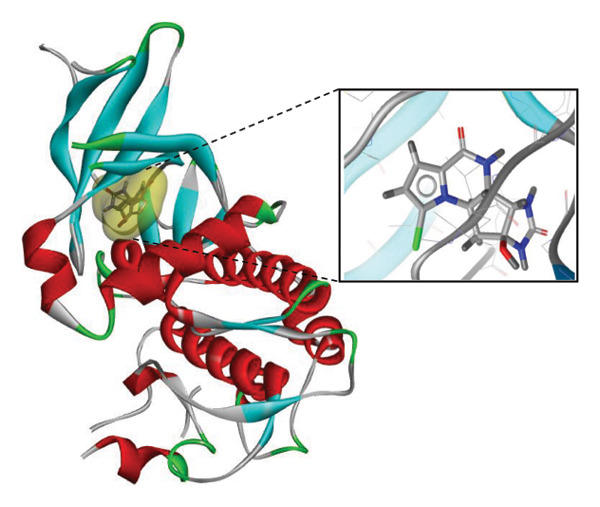
(d)

**Figure:**
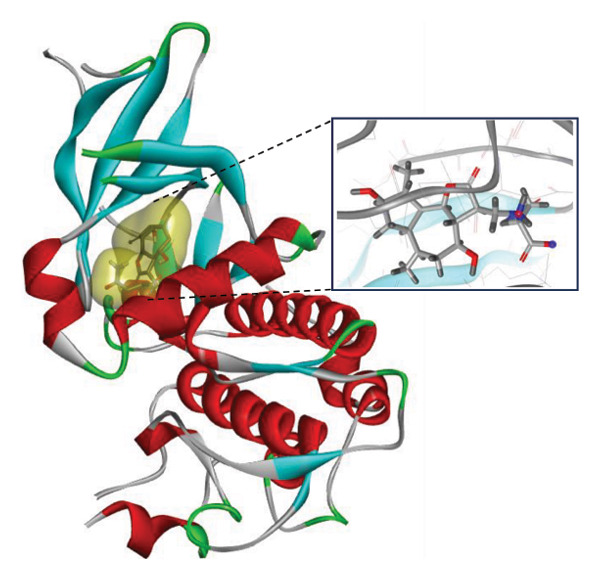
(e)

**Figure:**
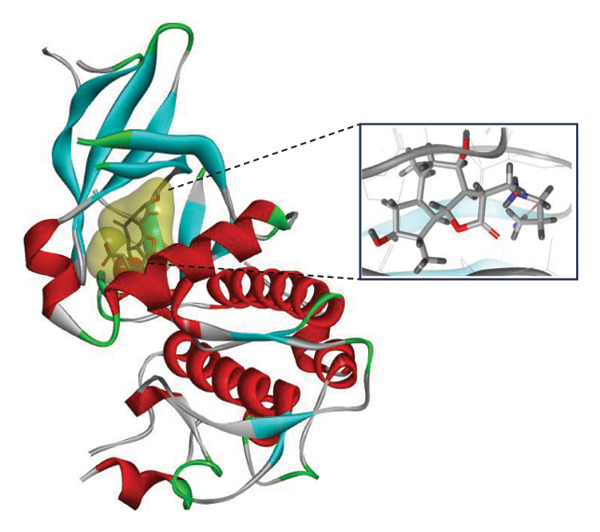
(f)

FIGURE 82D interaction diagrams of hit compounds docked with LKB1 protein ((a) CID 15000037, (b) CID 185971, (c) CID 9998735, (d) CID 177418, (e) CID 24862587, and (f) CID 24862637). The images have been generated using BIOVIA Discovery Studio.

**Figure:**
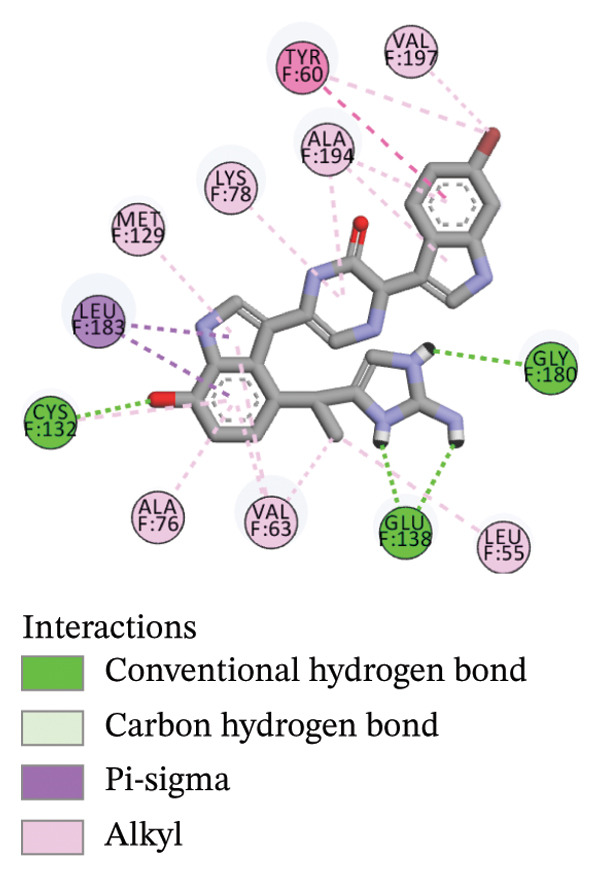
(a)

**Figure:**
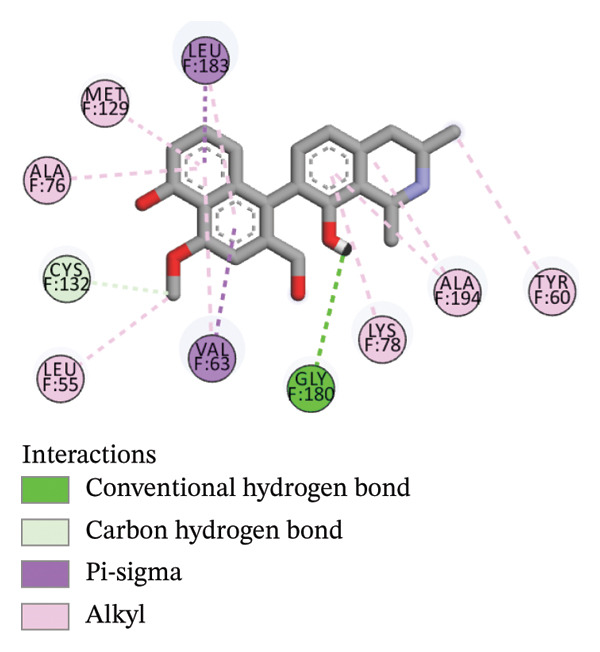
(b)

**Figure:**
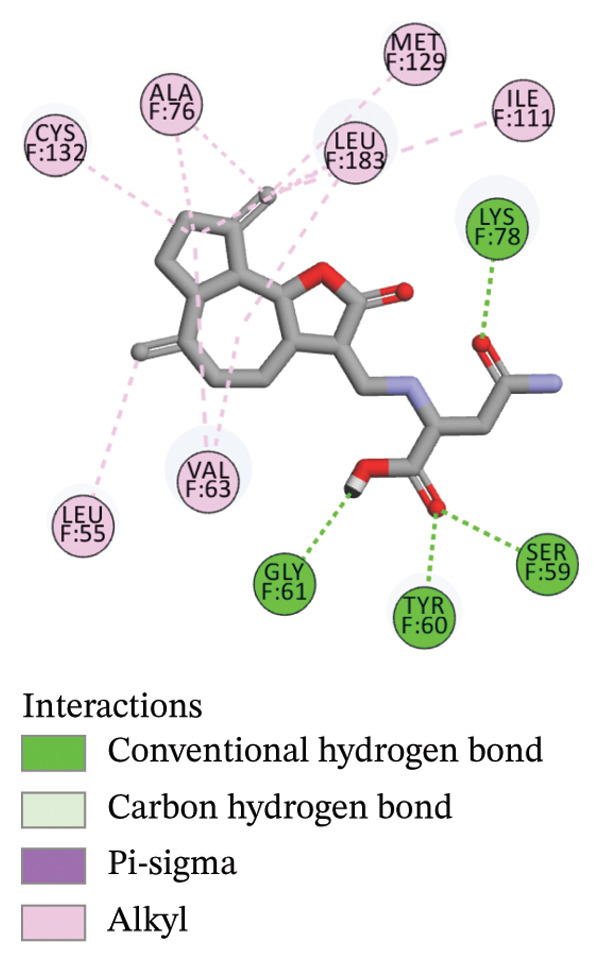
(c)

**Figure:**
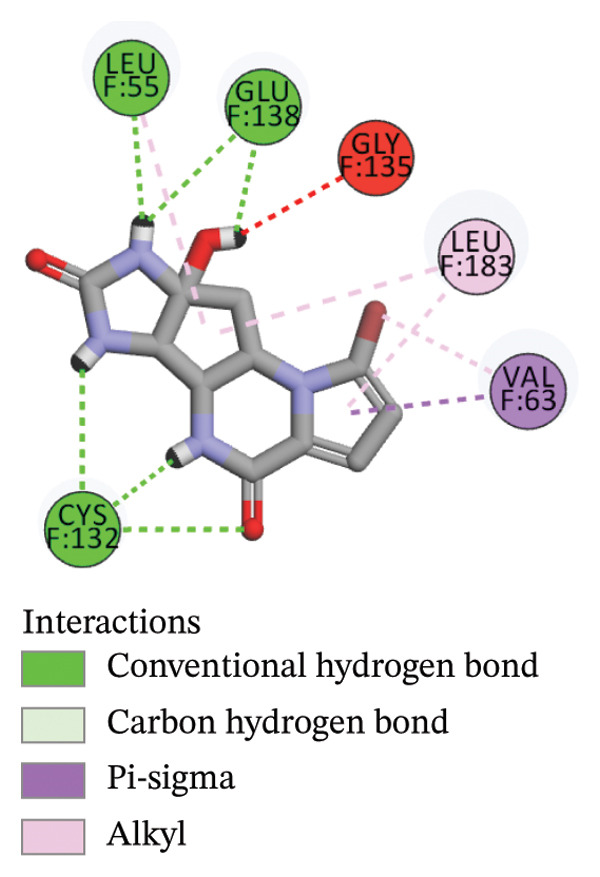
(d)

**Figure:**
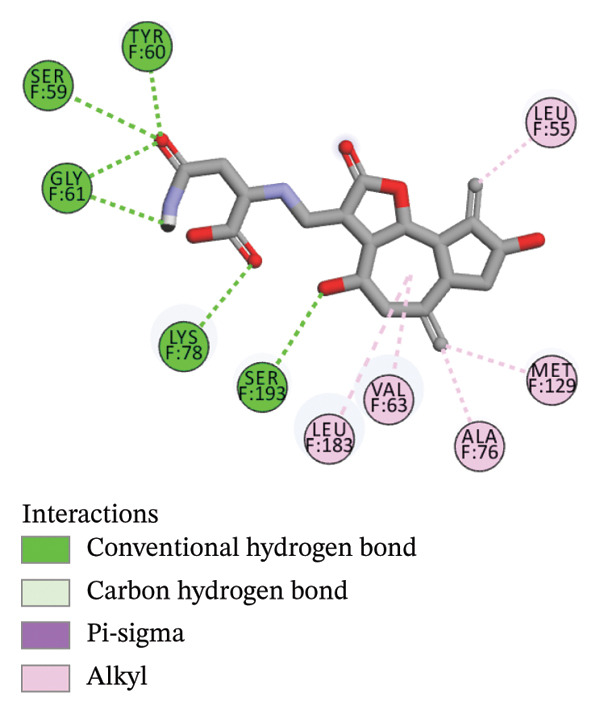
(e)

**Figure:**
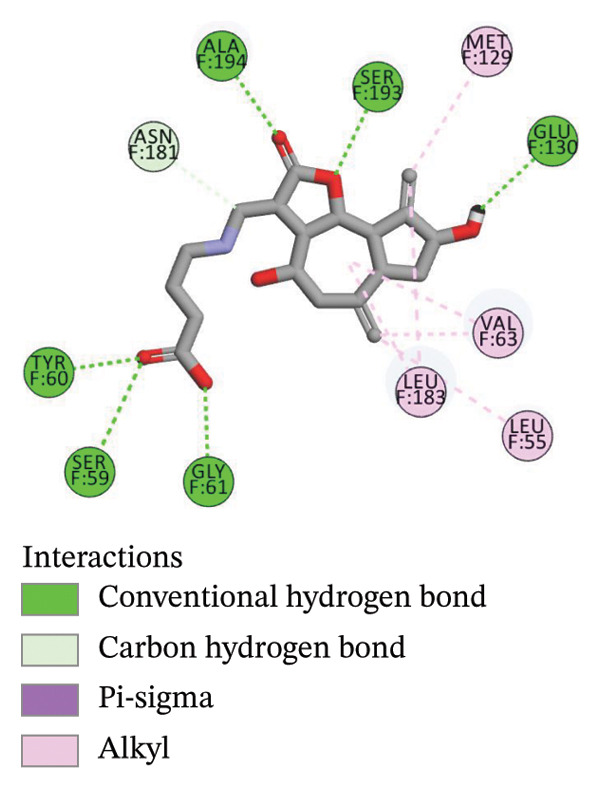
(f)

### 3.10. MD Simulation of Control Compound

MD simulation provides insights into the dynamic behavior and stability of protein–ligand interactions. Accordingly, a 100 ns MD simulation was performed for the control compound, phenformin, in complex with LKB1 to evaluate its stability and interaction profile under simulated physiological conditions. Figure 1 illustrates the RMSD and RMSF plots for the phenformin–LKB1 complex. Typically, RMSD and RMSF fluctuations within 1–3 Å are indicative of a stable protein–ligand complex.

The phenformin–LKB1 complex demonstrated strong stability, with RMSD values consistently ranging between 1 and 2 Å. Similarly, RMSF values remained below 3 Å, indicating minimal residue‐level fluctuations. These results suggest that the complex retained its conformational integrity throughout the simulation, confirming high structural stability. Therefore, the RMSD and RMSF profiles from both plots reflect robust protein binding and overall system stability. Figure 9 shows the 100 ns MD simulation data for phenformin–LKB1 complex.

FIGURE 9Molecular dynamics simulation 100 ns run data for phenformin–LKB1 complex showing (a) amino acid residue interaction, (b) RMSD plot, and (c) RMSF plot.

**Figure:**
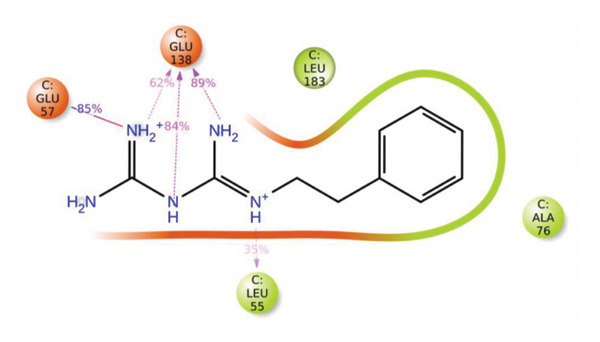
(a)

**Figure:**
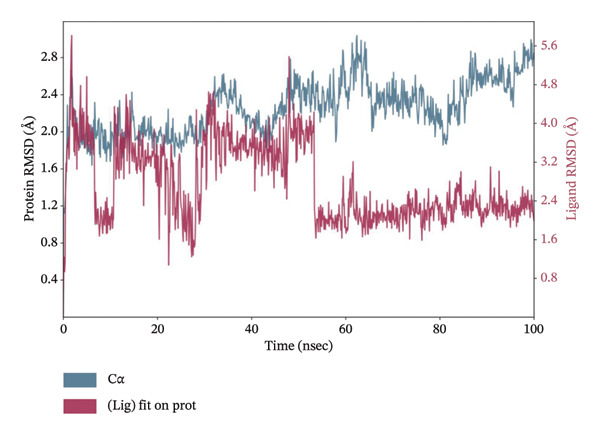
(b)

**Figure:**
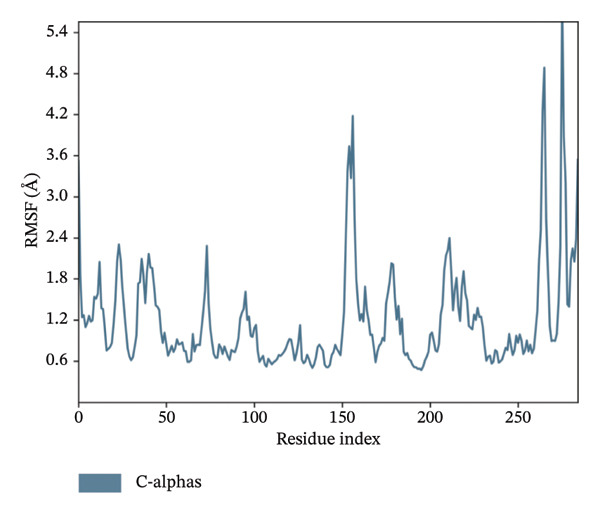
(c)

### 3.11. MD Simulation Studies of Top 4 Compounds With LKB1

MD simulations pave a pathway to analyze the dynamic behavior and stability of a protein–ligand complex under human body conditions. Therefore, an extended dynamics simulations of 100 ns of the filtered compounds with LKB1 were conducted to determine their stability and characteristics in a simulated environment of human body conditions [35]. Figure 10 shows the RMSD data plots for both the protein backbone and ligand during the whole simulation period for the top 4 compounds dragmacidin D, dioncopeltine A, saussureamine C, and agelastatin D. Small RMSD value fluctuations between 1 and 3 Å are usually used as a signifier for protein‐ligand stability [36].

FIGURE 10RMSD plots of molecular dynamics (MD) of target LKB1–drug complexes: LKB1 complexed with top four ligands (a) dragmacidin D, (b) dioncopeltine A, (c) saussureamine C, and (d) agelastatin D.

**Figure:**
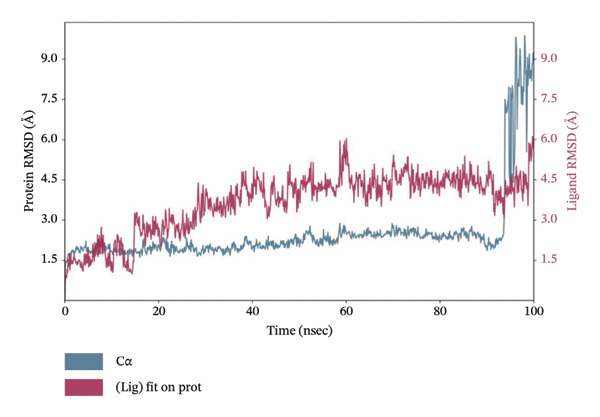
(a)

**Figure:**
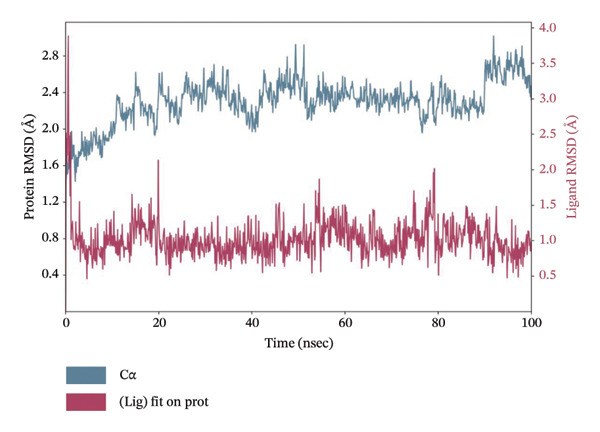
(b)

**Figure:**
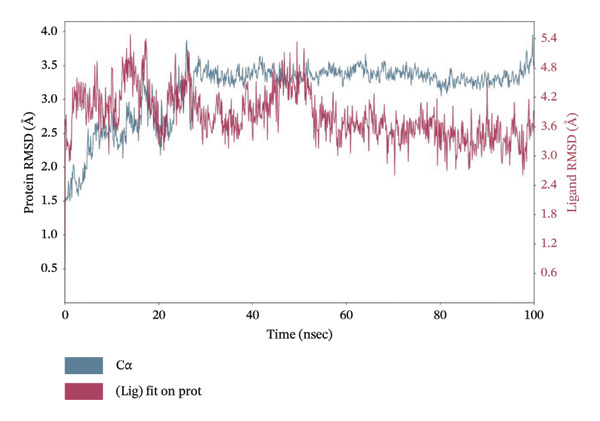
(c)

**Figure:**
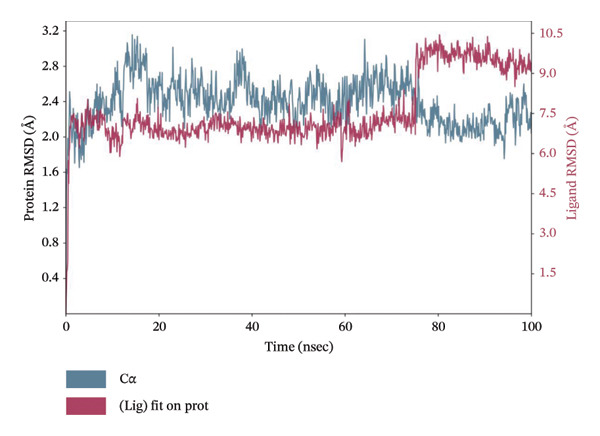
(d)

The RMSD plots of saussureamine C protein–ligand complex showed stability during the whole simulation period. No fluctuations were seen above the 3 Å range during the whole simulation period for both the protein backbone and ligand indicating excellent stability of the complex.

Dioncopeltine A–LKB1 complex also showed excellent stability without any RMSD values fluctuating over the 2 Å range during the whole 100 ns simulation period. The complex remained tightly bound with the ligand for the whole simulation period indicating excellent stability.

In a similar fashion, agelastatin D and LKB1 complex showed excellent stability without any RMSD fluctuation over 3 Å and the protein and ligand complex trajectory was superimposed indicating excellent stability.

Dragmacidin D–LKB1 complex protein RMSD showed slightly large fluctuations of about 5 Å during 93.8 nanoseconds but then protein backbone stabilized around 7–9 Å and continued this trend until the end of the simulation period. Ligand RMSD was stable throughout the whole simulation period of any major fluctuations. This indicates that the protein was tightly bound with ligand although the protein incurred some instability during that time period.

In MD simulation, RMSF indicates the local conformational changes of the protein backbone residues. Low RMSF value fluctuations within 1–3 Å indicate low conformational changes in the residues and overall protein stability. Figure 11 represents the RMSF values of residues for the top 3 ligands. In Figure 11, we can see that for dioncopeltine A, saussureamine C, and agelastatin D, there is only one almost identical high fluctuation peak in the 220–230 residue range. Besides this fluctuation, almost all the residues have high conformational stability without any fluctuations above 3 Å. So, for these 3 compounds, it can be said that the protein backbone has high stability. In the case of dragmacidin D, the RMSF values have very high fluctuation values in the residues after 220–2025. This might be the reason behind the periodic RMSD slight backbone fluctuation of dragmacidin D, as the residues locally conformed during the simulation period.

FIGURE 11RMSD plots of molecular dynamics (MD) of target LKB1–drug complexes: LKB1 complexed with top four ligands: (a) dragmacidin D, (b) dioncopeltine A, (c) saussureamine C, and (d) agelastatin D.

**Figure:**
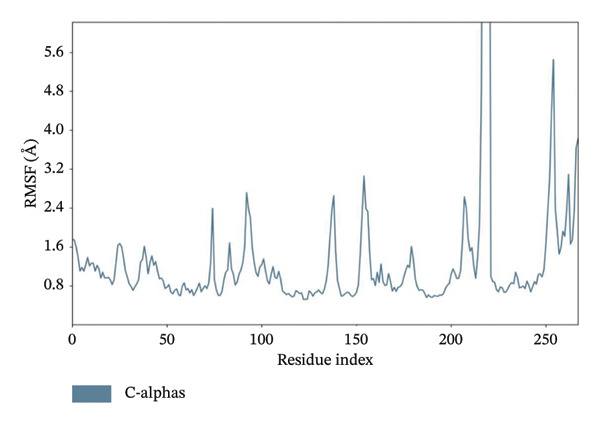
(a)

**Figure:**
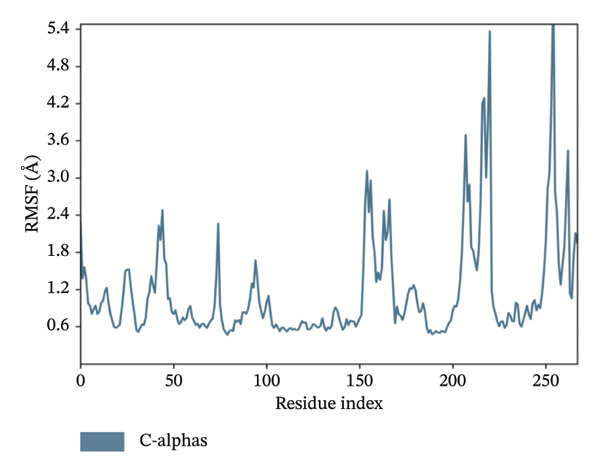
(b)

**Figure:**
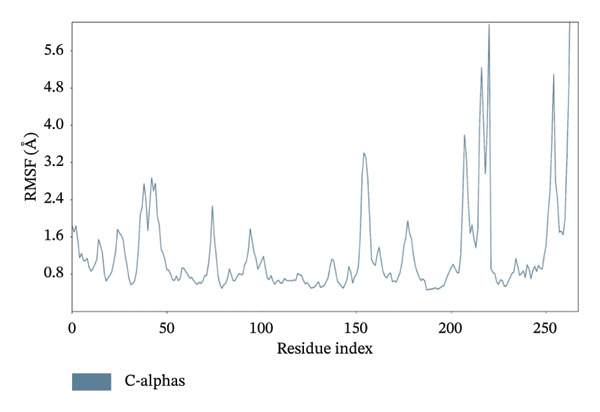
(c)

**Figure:**
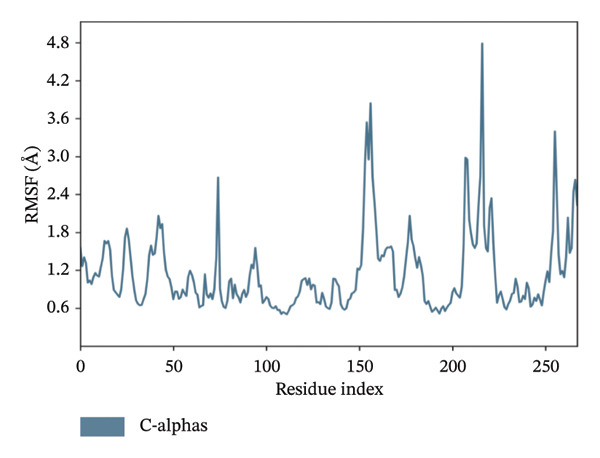
(d)

## 4. Discussion

AMPK is a key regulatory protein that is responsible for a wide range of metabolic pathways like metabolism of carbohydrates, lipids, amino acids, and cholesterol and other mitochondrial functions like autophagy and cell growth. It is commonly targeted as a direct approach of regulating glucose metabolism in diabetic patients by allosteric activation. However, developing new potent drugs which directly activate AMPK by allosteric binding has become quite challenging. That is why, researchers are considering LKB1 protein as an alternative target to indirectly activate the AMPK signaling to discover more potent, therapeutically efficacious drugs. LKB1 phosphorylates AMPK to indirectly activate this protein to regulate various metabolic functions including controlling glucose metabolism. Apart from AMPK, LKB1 can activate SNRK signaling pathway to induce glucose transport in skeletal muscle cells. A study provided evidence of the role of LKB1 in limiting inflammation‐driven metabolic dysfunction and insulin resistance by neutralizing IL‐17A via downstream AMPK and SIK signaling. Several studies have found that, when intracellular ATP levels drop and AMP levels rise, as occurring during hypoxia or nutrient restriction, AMPK is triggered [37]. When AMPK is turned on, it promotes catabolic processes, boosts cellular glucose uptake, and suppresses anabolic activities. Another study showed that activation of AMPK increases glucose absorption, glucose transporter 4 (GLUT4) expression, and mitochondrial biogenesis under metabolic stress [38]. It has also been found that skeletal muscle glucose transport increases in response to AICAR activation of AMPK without being dependent on insulin and this occurs simultaneously with the GLUT4 being translocated to the skeletal muscle cells’ plasma membrane [39]. All of these studies point to the possibility that activating AMPK can also activate skeletal muscle’s glucose transport. Furthermore, it has been discovered that the polarity, size, total mass, and glucose‐stimulated insulin secretion (GSIS) of pancreatic β cells are influenced by the LKB1‐mediated AMPK pathway [40]. Located on chromosome 19p13.3, the LKB1gene, also called serine/threonine kinase 11 (STK11), contains 10 exons that are 23 kb long [41]. Thus, agonists of LKB1 protein can be beneficial in activating AMPPK‐mediated glucose utilization cascade.

In the present study, saussureamine C is one of the top ligands with good binding affinity towards LKB1. It is an amino acid adduct of sesquiterpene lactones, which was first isolated from the dried root of *Saussurea lappa* Clarke known as Chinese Saussureae Radix [42]. Saussureae Radix was originally a Chinese herbal medicine used as an aromatic stomachic whose efficacy has been proved by a study showing that saussureamine C can exhibit antiulcer effects on HCl/ethanol‐induced lesions in rats’ gastric mucosa [43]. However, in the case of inflammation inhibition, saussureamine C showed little to no activity unlike other amino‐acid conjugates of sesquiterpene lactones like saussureamine A and saussureamine B [44]. Another research found that saussureamine C has high antineoplastic properties manifested as stimulation of apoptosis of colorectal and breast cancer cells [45]. Further research is required to find out more about its pharmacological properties apart from the aforementioned ones [46].

Pulchellamine B is a secondary metabolite of the plant genus *Saussurea*; pulchellamine B is an amino acid and sesquiterpene lactone conjugate [47]. It was initially extracted using methanol from *Saussurea pulchella*’s aerial parts. In Korean traditional medicine, *Saussurea pulchella* has been utilized for its biological properties, which include anti‐inflammatory, antihypertensive, antihepatitis, and antarthritic properties [48]. Recent pharmacological studies have shown that both the sesquiterpenes and the ethanol extract from *Saussurea pulchella* have pronounced anti‐inflammatory properties. Dragmacidin D is a heterocyclic bisindole alkaloid primarily separated from a deep‐water marine sponge of the genus Spongosorites [49, 50]. This compound exhibits antimicrobial activity against *Escherichia coli*, *Bacillus subtilis*, *Pseudomonas aeruginosa*, *Candida albicans*, and *Cryptococcus neoformans* and antiviral activity against the feline leukemia virus (FeLV). It was also shown in research that this compound induces cytotoxicity in the P388 murine leukemia cell line and A549 human lung adenocarcinoma cell line [50]. It was also found that this compound can inhibit serine‐threonine protein phosphatases (PP) with a moderate potency and can also inhibit the neural nitric oxide synthase (bNOS). Besides, dragmacidin showed an 89.6% reduction in resiniferatoxin‐induced inflammation in the mouse ear edema model at 50 μg/ear dose [51].

Agelastatin D is a part of the cytotoxic pyrrole‐imidazole alkaloids family having a unique tetracyclic configuration with four adjacent stereogenic centers on the carbocyclic C‐ring [52]. It was initially separated in 1998 from the sponge *Cymbastela* sp. native to the Indian Ocean. It was found to show anticancer effects by inducing apoptotic cancer cell deaths and arresting cell growth in the G2/M phase of the cell cycle, without affecting tubulin dynamics within cells [36, 53]. Pulchellamine A is an amino acid‐sesquiterpene lactone conjugate isolated from the methanol extract of the aerial parts of *Saussurea pulchella*, a Korean medicinal plant. Though the pharmacological effects of this compound are still unexplored, the source plant was widely distributed in Korea and traditionally used as medicine for the treatment of inflammation, hypertension, hepatitis, and arthritis [54].

Some recent studies have discovered a couple of new agents that may help battle T2DM. For example, antroalbol H, which is obtained from the basidiomycete mushroom *Antrodiella albocinnamomea*, controls hyperglycemia by facilitating Thr‐189 phosphorylation of LKB1, which in turn activates AMPK and finally increases cellular glucose intake [7]. Another study has found two novel AMPK activators named Xn and Xc, which enhance glucose homeostasis when used in very small doses. It has been verified that elevated GLUT4 translocation led to higher glucose absorption via LKB1‐dependent AMPK activation in vitro and more desirable glucose tolerance via AMPK activation in skeletal muscle in vivo. The fact that the molecular target of antroalbol H is LKB1 provides evidence that the pathway can prove to be an effective drug therapeutic target for designing new antidiabetic agents. The investigations on possible role of LKB1 in diabetes mellitus can be helpful for establishing a molecular target that can aid in drug design studies. Some of the proposed ligands such as saussureamine C and agelastatin D have good binding affinities towards LKB1; ADMET properties and ligand–protein stable complexes can be further studied to develop new antidiabetic medications.

## 5. Conclusion

Currently, 537 million adults have diabetes; by 2030, that number is predicted to increase to 643 million. It is essential to focus on creating novel therapeutic approaches in order to prevent and treat this illness. In the fight against type 2 diabetes, AMPK and LKB1 are attractive targets, especially LKB1, whose hypoglycemic properties are still poorly understood. Thus, there is ample opportunity for further research on this topic. The current study’s screening of 29,000 natural phytochemicals generated a shortlist of hit compounds that, by acting as agonists of the LKB1‐mediated pathway, may benefit diabetes patients. In vitro analyses using the suggested hits could not be conducted due to resource constraints. However, future studies using proposed compounds may be helpful in discovering new leads that will be beneficial in controlling the symptoms of T2DM. Research on the potential involvement of LKB1 in diabetes mellitus may be useful in identifying a molecular target for studies on medication development. To create new antidiabetic drugs, more research can be done on some of the suggested ligands, such as saussureamine C and agelastatin D, which have strong binding affinities towards LKB1, acceptable ADMET characteristics, and ligand–protein stable complexes.

**Nomenclature**
LKB1: Liver kinase B1
AMPK: AMP‐activated protein kinase
ADMET: Absorption, distribution, metabolism, excretion, and toxicity
CADD: Computer‐aided drug development design
PDB: Protein Data Bank
RO5: Lipinski’s rule of five
MDS: Molecular dynamics simulation
Rg: Radius of gyration
RMSD: Root mean square deviation
RMSF: Root mean square fluctuation

## Author Contributions

Rumman Reza, Md. Nazmus Samdani, and Niaz Morshed contributed to idea generation and manuscript writing. Md. Nazmus Samdani, Niaz Morshed, and Rumman Reza performed the docking analyses and generated the data. Niaz Morshed curated MD simulation data. Raihana Haque Jebin and Imtiaz Ahmed contributed to manuscript writing. Md. Selim Reza supervised the work, reviewed the manuscript, and edited the draft manuscript.

## Funding

No funding was received for this manuscript.

## Conflicts of Interest

The authors declare no conflicts of interest.

## Supporting Information

Additional supporting information can be found online in the Supporting Information section.

## Supporting information


**Supporting Information 1** Supporting table 1: binding affinity (in kcal/mol) of ligands with good pharmacophore fitting score docked with liver kinase B1 protein.

**Supporting Information 2** Supporting table 2: all the parameters of ADMET profile as retrieved from SwissADME of top compounds with high binding affinity towards liver kinase B1 protein.

**Supporting Information 3** Supporting figure 1: docked poses of reference PDB and redocked PDB of LKB1 with bound ligand.

**Supporting Information 4** Supporting figure 2: oral bioavailability radar images of control compounds (metformin, phenformin, and buformin).

## Data Availability

The data that support the findings of this study are available in the supporting information of this article.
