# Supplementary figures and images for: Ligand‐Based Pharmacophore Mapping and Virtual Screening for the Search of Biguanide‐Like Molecules With Antidiabetic Potentials Targeting Liver Kinase B1
Source: Biochem Res Int. 2026 Feb 19;2026:8369459. doi: 10.1155/bri/8369459 (PMC12917864; doi:10.1155/bri/8369459)

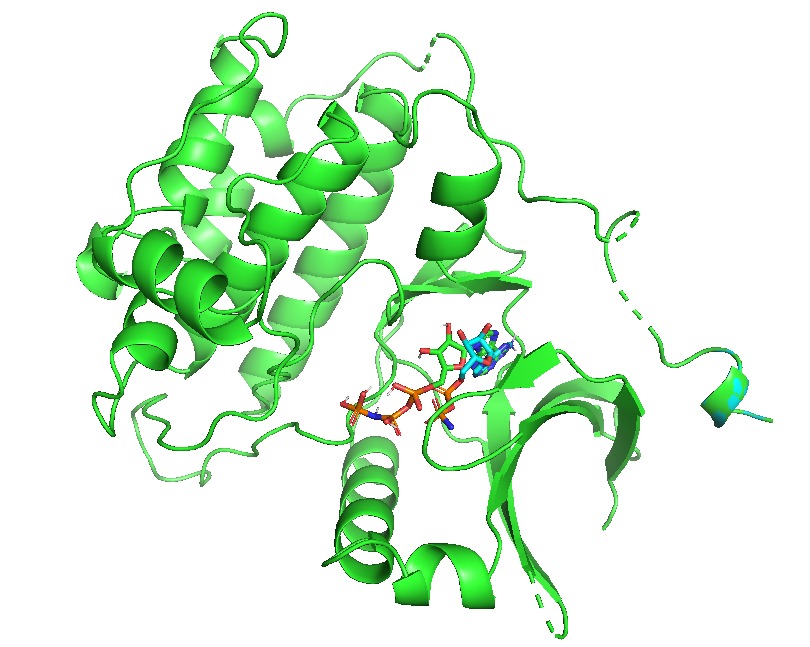


**Supplementary figure 1.** Docked poses of reference PDB and re-docked PDB of LKB1 with bound ligand.

Supplement: Supplementary file 3 — Supporting Information 3 Supporting figure 1: docked poses of reference PDB and redocked PDB of LKB1 with bound ligand. [file BRI-2026-8369459-s003.docx]

Supplementary figure 2


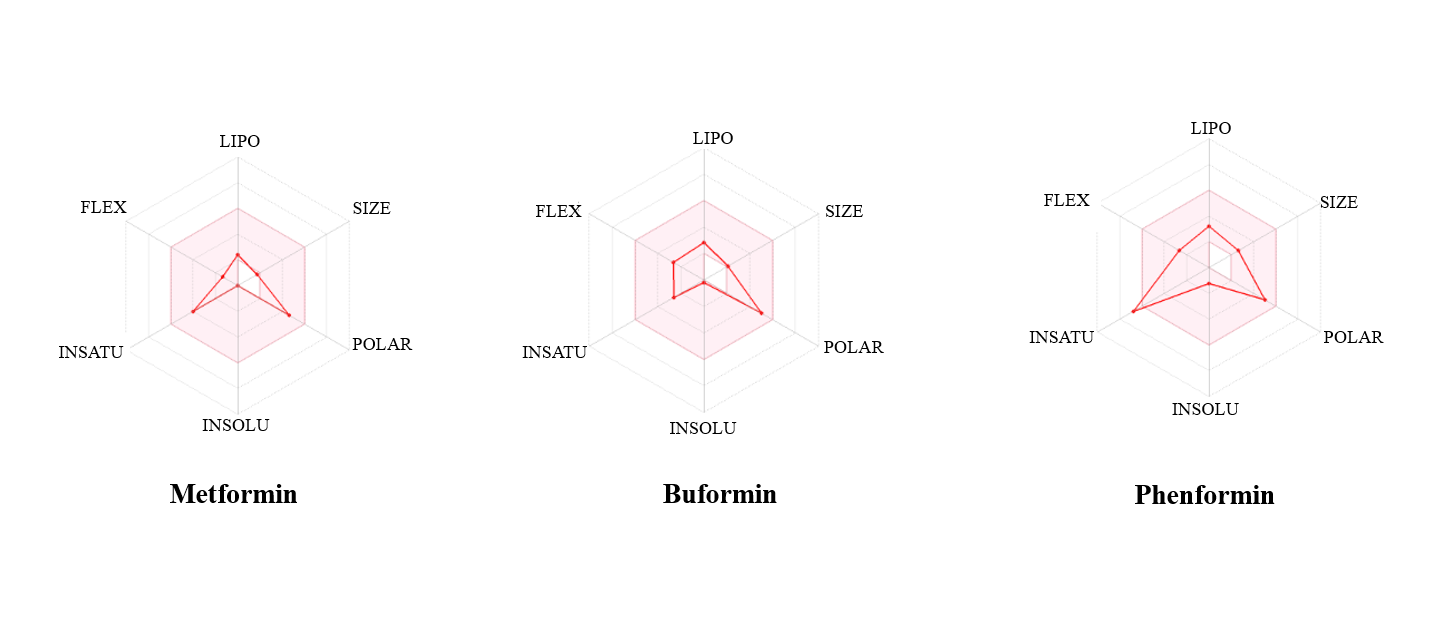

Supplement: Supplementary file 4 — Supporting Information 4 Supporting figure 2: oral bioavailability radar images of control compounds (metformin, phenformin, and buformin). [file BRI-2026-8369459-s002.docx]
